# Post paracentesis deep circumflex iliac artery injury identified at angiography, an underreported complication

**DOI:** 10.1186/s42155-019-0068-y

**Published:** 2019-07-19

**Authors:** Jalil Kalantari, Mark H. Nashed, Jason C. Smith

**Affiliations:** Department of Interventional Radiology, Loma Linda University Medical Center, 11234 Anderson Street, Loma Linda, CA 92354 USA

**Keywords:** Paracentesis, Complication, Embolization, Deep circumflex iliac artery

## Abstract

**Background:**

Though injury to the inferior epigastric artery (IEA) is reported to be the most common source of hemorrhagic complications from paracentesis, we wish to present our experience involving deep circumflex iliac artery (DCIA) injuries that in our experience is the artery most frequently injured during paracentesis.

**Methods:**

Sixteen patients with clinically significant hemorrhage following paracentesis were referred to our Interventional Radiology service for trans-catheter embolization. Patterns of hemorrhage from diagnostic cross-sectional imaging and subsequent angiographic findings and management were investigated.

**Results:**

8/16 patients (50%) had angiographic evidence of injury to the DCIA and 4/16 patients (25%) had evidence of injury to the IEA, with two of these patients demonstrating hemorrhage from both the DCIA and IEA; 3/16 patients had injuries to subcostal and/or intercostal arteries; while 3/16 patients had negative angiograms. All patients underwent embolization of the identified injured arteries, and empiric embolization was performed of the DCIA and/or IEA in the three patients with negative angiograms. Fourteen of sixteen patients stabilized post embolization, while two patients required a second embolization procedure to achieve hemostasis; all patients were subsequently discharged home in stable condition.

**Conclusion:**

Both the IEA and the lesser known DCIA need to be considered when performing paracentesis and at subsequent angiography for post paracentesis iatrogenic hemorrhage. Knowledge of both of these at-risk abdominal wall arteries may help minimize hemorrhagic complications from paracentesis.

## Introduction

Paracentesis related iatrogenic hemorrhage necessitating treatment is rare, occurring in 1% of all paracenteses (Mallory and Schaefer 1978; Runyon 1986). Practitioners are taught to needle the peritoneum laterally in the lower quadrants where the largest fluid pockets are located (Thomsen et al. 2006; Sakai et al. 2005). This approach avoids the inferior epigastric artery (IEA) coursing within the rectus sheath, which is reported to be the most commonly injured vessel during paracentesis that results in clinically significant hemorrhage necessitating endovascular treatment (Sobkin et al. 2008). Interestingly, few cases have been reported of paracentesis related bleeding from the deep circumflex iliac artery (DCIA) (Day et al. 2014; Satija et al. 2012; Kang et al. 2012), which in our experience is the most commonly injured artery from paracentesis subsequently identified at angiography. We wish to present these cases, review the anatomy relevant to the inferior epigastric and deep circumflex iliac arteries, and discuss how to possibly minimize the risk of injury from paracentesis to this lesser known artery.

## Materials and methods

Our institutional review board approved this retrospective study that includes 16 patients identified through a data mining software (Montage-Nuance Communications, Burlington, MA, USA) who were referred to our interventional radiology service for embolization of hemorrhage after paracentesis between 2007 and 2018. Most patients had clinically indicated diagnostic imaging examinations as part of the work up for suspected hemorrhage. Clinical and procedural data for both paracenteses and subsequent angiographic studies were obtained via the patients’ electronic medical record and departmental picture archival system.

Briefly, patients underwent diagnostic angiography under moderate sedation. Via a transfemoral approach (either ipsi- or contra-lateral, at the discretion of the interventionalist), external iliac arteriography of the ipsilateral side as the suspected site of bleeding from recent paracentesis was performed in standard fashion. A three French micro-catheter was then advanced in co-axial fashion into the specific abdominal wall artery where hemorrhage was identified, and embolization was performed in standard fashion per the operators’ discretion with some combination of polyvinal alcohol particles (355–500 um Contour™ embolization particles, Boston Scientific, Cork, Ireland), Gelfoam®pledgets (Pharmacia UpJohn Company, Kalamazoo, MI, USA), off-label use of liquid Onyx (MicroTherapeutic, Inc., ev3™, Irvine, CA, USA), and/or platinum microcoils. When the angiogram was negative for contrast extravasation or psuedoaneurysm and a high clinical suspicion of active bleed remained, empiric embolization of the arterial supply to the region of interest was performed. Hemostasis was generally obtained by placement of an AngioSeal™ vascular closure device (Terumo Medical Corporation, Somerset, NJ, USA). The patients were monitored on the clinical floor until both hemodynamic status and hemoglobin stabilized.

## Results

This study included a total of 16 patients, 11 males and 5 females, with a mean age of 55 years (Table 1). Thirteen patients (82%) had underlying cirrhosis secondary to alcohol (38%), hepatitis C virus (38%), or primary biliary cirrhosis (6%); one patient had ascites secondary to cardiomyopathy (6%), another had ascites secondary to septic shock (6%), and one patient had ascites due to cholangiocarcinoma (6%).

**Table 1:** Patient demographics (*n* = 16)

Characteristics	N (%)
Gender	
Male	11
Female	5
Age, mean (years)	55
Cirrhosis diagnosis	13 (81%)
DCIA injury	8 (50%)
IEA	5 (31%)
Other injuries	4 (25%)
Repeat embolization	2 (12%)
Embolization type	
Particles & Coils & Onyx	1 (6.2%)
Particles & coils	5 (31%)
Particles & Gelfoam	1 (6.2%)
Particles only	2 (12.5%)
Coils only	6 (37.5%)
Gelfoam	1 (6.2%)

Paracenteses were performed by hospitalists (*n* = 10, 63%) or interventional radiologists (*n* = 3, 19%), and in three cases no procedure note was found. The paracenteses were performed under ultrasound guidance in all documented cases, eight cases of right lower quadrant access and five cases of left lower quadrant access. The patients at the time of the paracentesis had an average prothrombin time of 24.2 seeconds (*range = 12–43.4*), platelet count of 106.6 × 10^3^/μL blood (*range = 45–230*), and body mass index (BMI) of 28.5 Kg/m^2^ (*range = 19–46*). An average of 4.6 L of ascites (*range = 1.2–8 L*) was collected, with all but two cases documenting a non-sanguineous aspirate. In three cases, prolonged oozing at the puncture site was noted, and manual pressure was applied for 5–10 min at the time of paracentesis.

The average time between paracentesis and diagnosis of bleeding as well as the average time to perform angiography were 0.8 day (*range = 0–3*) and 3 days (*range = 0–15*), respectively. The most common presenting symptom was abdominal pain and in some cases was altered mental status with hypotension, with one case requiring a rapid response code activation. All patients received medical management including administration of blood products prior to decision to proceed to angiography.

**Fig. 1:**
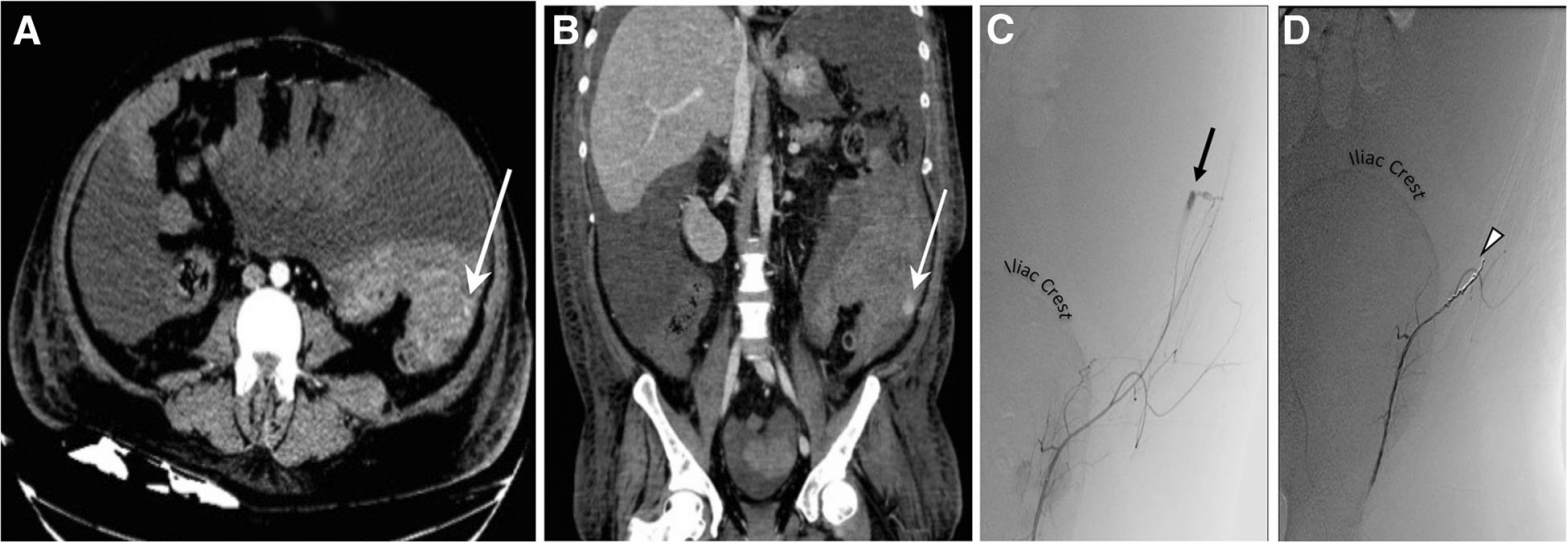
**a** Axial and **b** coronal CT images demonstrate ascites with fluid-fluid level suggestive of blood products with active contrast extravasation along the left lateral abdomen (arrows). Subsequent digital subtraction angiography images show extravasation **c** from an injury to a branch of the left deep circumflex iliac artery (arrow), and **d** the same patient after coil (arrowhead) and Gelfoam embolization of the vessel indicating successful occlusion of the artery and resolution of hemorrhage

Fourteen out of sixteen patients had diagnostic computed tomography (CT) prior to angiography, 1 patient had a diagnostic ultrasound only, and 1 patient was transferred emergently to interventional radiology without imaging. Signs of hemorrhage were identified in all imaged patients and included variable findings of abdominal wall hematoma, hemoperitoneum, and/or pseudoanurysm, with or without active extravasation (Fig. 1a, b).

Of the 16 initial angiograms, 8 patients (50%) had angiographic evidence of injury to the DCIA (Fig. 1c, d) and four patients (25%) had evidence of injury to the IEA, with two of these patients demonstrating hemorrhage from both the DCIA and IEA; in three patients, the injured arteries were noted to be subcostal or intercostal arteries, including one patient who had had her DCIA embolized at her first embolization procedure; finally, no evidence of arterial injury could be identified in three patients. In the 13 patients where an injured vessel was identified on initial angiography, trans-catheter embolization was performed of these vessels. Of the three patients without angiographic evidence of arterial injury, empiric embolization of both IEA and DCIA was performed in two patients and only the IEA in one patient. In total, 10 out of the 16 patients had embolization performed of the DCIA.

Fourteen patients (88%) stabilized post embolization, while two patients (12%) who initially had embolization performed of the DCIA required repeat embolization because of continued dropping hemoglobin. In the first case, repeat angiography demonstrated prominent collateral flow from lumbar and intercostal arteries to the paracentesis site, and embolization of these vessels was performed. In the second case, contrast extravasation from the IEA was noted, which was then embolized successfully. There were no documented procedural complications. All patients were subsequently discharged home in stable condition.

## Discussion

Bleeding complications from paracenteses are rare, occurring 1% of the time (Runyon 1986). However, these can result in life-threatening hemorrhage and carry significant morbidity and mortality. Patients are frequently coagulopathic due to intrinsic liver disease, as with the majority of the patients in our report.

Proceduralists have been taught to minimize the bleeding risk and maximize fluid acquisition by preferentially targeting the left lower abdominal quadrant based on anatomic landmarks 2–4 cm cephalad and medial to the anterior superior iliac spine which is sufficiently lateral to the rectus sheath to avoid the risk of injury to the IEA (Sakai et al. 2005). Several factors may distort the normal anatomy, thus making these landmarks difficult to determine in everyday practice: 1) In patients with ascites, the abdominal musculature and IEA are stretched laterally (Sharzehi et al. 2014), and 2) With obesity, the body habitus may also obscure anatomic landmarks such as the anterior superior iliac spine, as with many of the patients in our report. However, the increased utilization of ultrasound has allowed proceduralists to target the largest pocket of fluid regardless of anatomic location. It is worth noting that while the supra-pubic, midline approach is still performed by some so as to minimize risk of injury to the IEA, the majority of patients undergoing paracentesis have cirrhosis related portal hypertension, and lacerated varices along the peritoneal surface are relatively common in the peri-umbilical region; approximately one third of hemorrhagic complications from paracentesis may be due to these mesenteric varices (Sharzehi et al. 2014).

Injuries to the IEA have a relatively early presentation compared to injuries of other abdominal wall vessels (such as the DCI and varices) given the formation of a hematoma within the rectus muscle sheath compared to the lateral abdominal wall and the peritoneal cavity (Day et al. 2014; Rimola et al. 2007). Therefore, DCIA bleeding may be more difficult to identify compared to inferior epigastric bleeding, as it less likely to form a palpable abdominal wall hematoma (Day et al. 2014). When this happens, occult hemoperitoneum rather than visible abdominal wall hematoma is the main hemorrhagic feature, and delayed presentation of clinically significant bleeding up to 4 days later may occur and present a diagnostic and management challenge (Arnold et al. 1997). In our series, only 9 of the 16 patients developed hematomas by physical exam or by cross-sectional imaging, and 4 of the 8 patients with angiographically documented injured DCIAs presented with hemoperitoneum alone. Traditionally it has been recommended to follow patients at risk for bleeding after paracentesis closely including serial daily hemoglobin checks for several days (Arnold et al. 1997; Katz et al. 2013). However, this may not be pragmatic in contemporary outpatient practice, as patients are routinely scheduled as outpatients for large volume paracenteses.

The mainstay of treatment is first recognition, followed by medical management, and then embolization for patients not responding to conservative management. To date, the largest report of embolization for post paracentesis bleeding described 19 patients with the IEA as the bleeding source in the majority of their patients, none of whom had evidence of injury to the DCIA (Sobkin et al. 2008). If an infraumbilical approach was taken and an injury to the recannalized paraumbilical vein occurs, arteriography will be negative. In a review of the literature, we were only able to identify five definitive cases of injury to the DCIA from paracentesis (Day et al. 2014; Satija et al. 2012; Kang et al. 2012; Rimola et al. 2007), of which four necessitated embolization procedures (one patient responded to conservative management alone). Other investigators have referenced the DCIA as an unusual culprit in abdominal wall hemorrhage from a variety of etiologies; these include Park et al. (Park et al. 2011) who reported embolizations in 12 patients for abdominal wall hemorrahge, including 11 IEAs and only 1 DCIA, though the authors made no mention as to the etiologies of the bleeds, and Mukind et al. (Mukund et al. 2018) who had 23 patients post paracentesis with mostly IEA injuries, but at least one patient with injury to the DCIA documented at angiography. However, in our experience, DCIA injury is the most commonly injured artery (50%) from paracentesis identified at angiography. As many hemorrhages are managed non-operatively, it is almost certain that the true incidence of injury to the DCIA is significantly underreported.

**Fig. 2:**
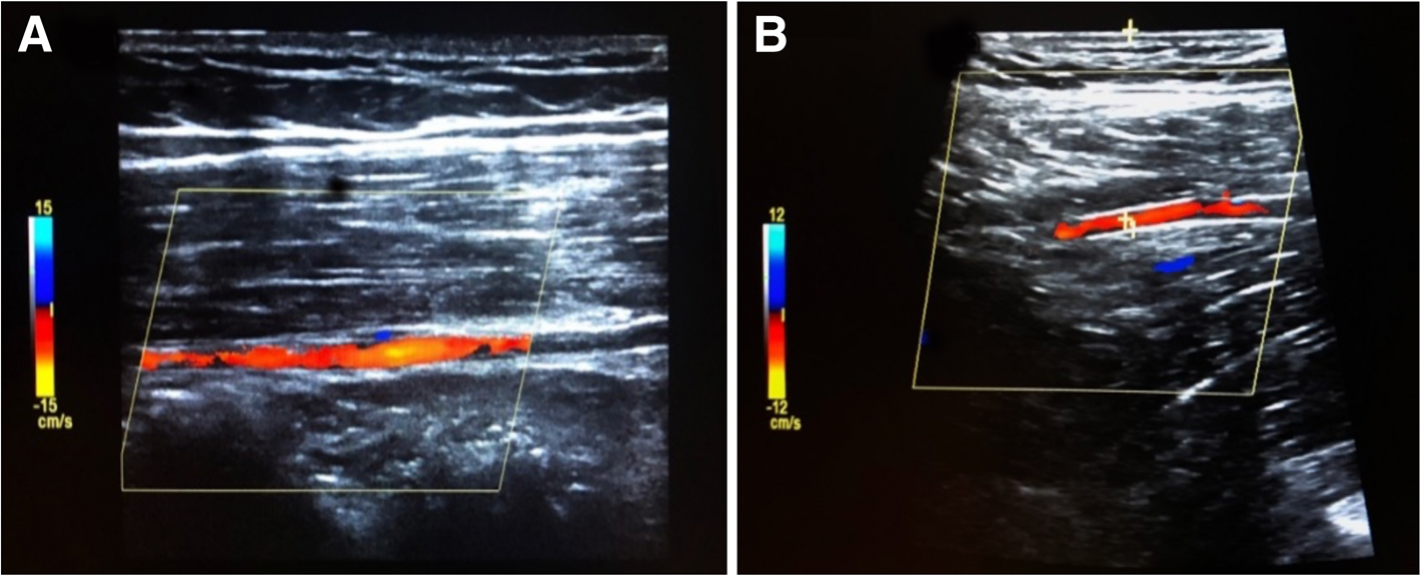
Mapping the **a** right inferior epigastric artery (IEA) and **b** deep circumflex iliac artery (DCIA) in a healthy volunteer using Doppler ultrasound

**Fig. 3:**
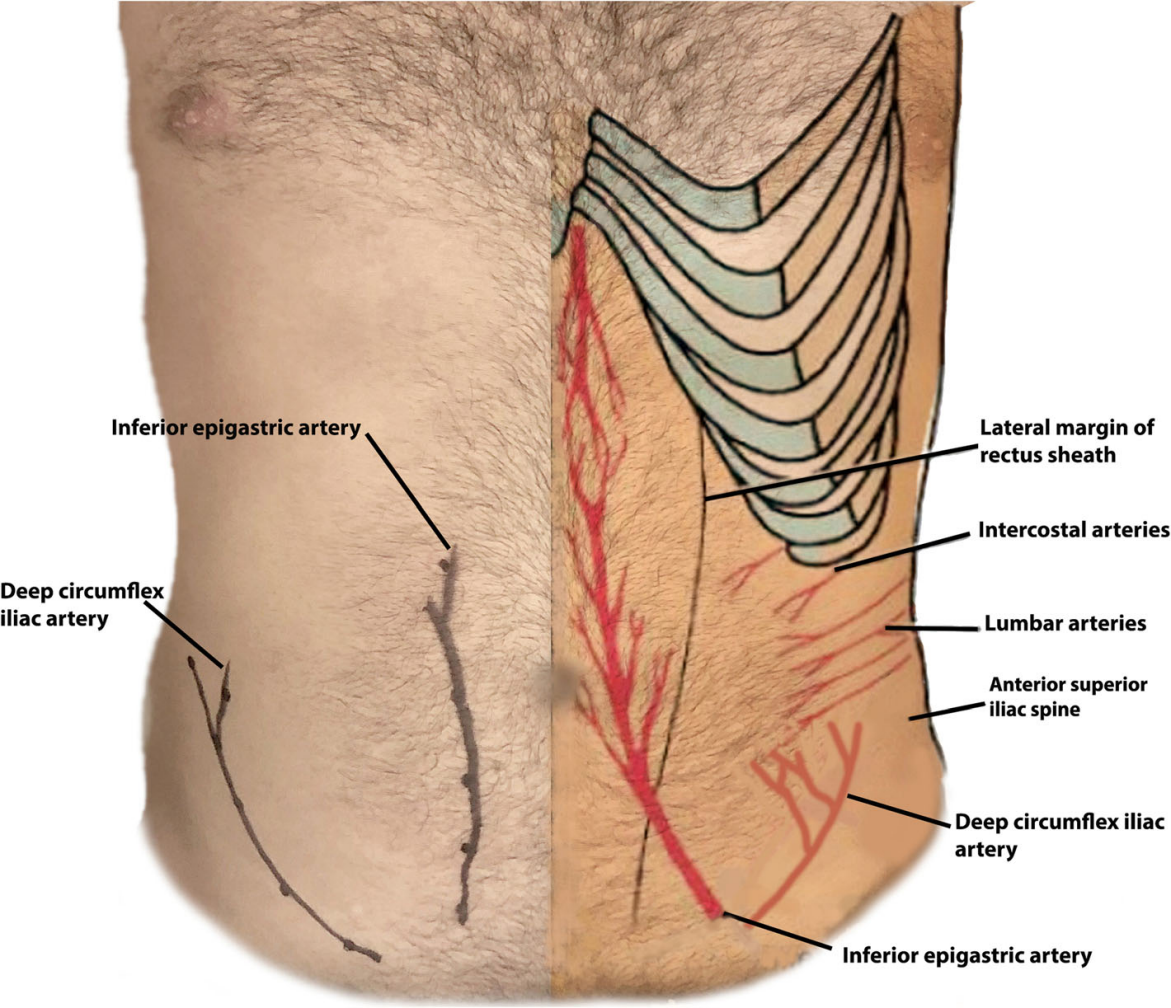
Anatomic drawing of important anterior abdominal wall arteries superimposed on a healthy volunteer with the right hemi-abdomen demonstrating the course of the arteries obtained by Dopper ultrasound images, see Fig. 2

We hypothesize that in consciously avoiding the IEA, practitioners choose a puncture site that is too lateral along the iliac crest which often cannot be easily palpated through a distended abdomen, and where the DCIA courses, resulting in injury to this vessel. While ultrasound evaluation of the peritoneum is primarily utilized to find the largest fluid pocket, mapping out the IEA with the highest frequency ultrasound transducer possible and palpating the iliac crest prior to paracentesis may help to select an appropriate “middle ground” between the two at-risk arteries (Stone and Moak 2015; Sekiguchi et al. 2013). Additionally, portal-systemic varices should be attempted to be identified and avoided. On thinner patients, the DCIA may even sometimes be identified on ultrasonography (Figs. 2 and 3). However, as both Sobkin (Sobkin et al. 2008) and Mukin (Mukund et al. 2018) have noted, as well as in our own experience, the injured artery is typically a small branch rather than the parent artery.

Limitations of our report included its retrospective nature, small patient group, and bias that only the most symptomatic patients were likely referred to IR with lack of more information regarding the patients with post paracentesis hemorrhage managed only conservatively at our institution over the reported time period.

## Conclusion

The most common, albeit rare, complication from paracentesis in this typically coagulopathic patient population is hemorrhage. Our reported cases illustrate that the DCIA needs to be considered alongside the IEA when performing paracentesis and at subsequent angiography for post paracentesis iatrogenic hemorrhage. Knowledge of both of these at-risk abdominal wall arteries may help minimize clinically significant iatrogenic hemorrhage from paracentesis and facilitate subsequent embolization when required.

## Data Availability

All data generated or analysed during this study are included in this published article.

## Abbreviations

BMI: Body mass index
CT: Computed tomography
DCIA: Deep circumflex iliac artery
IEA: Inferior epigastric artery
INR: International normalized ratio
